# Wearable Technology to Detect Motor Fluctuations in Parkinson’s Disease Patients: Current State and Challenges

**DOI:** 10.3390/s21124188

**Published:** 2021-06-18

**Authors:** Mercedes Barrachina-Fernández, Ana María Maitín, Carmen Sánchez-Ávila, Juan Pablo Romero

**Affiliations:** 1 Programa en Ingeniería Biomédica (PhD), ETSI Telecomunicación, Universidad Politécnica de Madrid (UPM), Avenida Complutense, 30, 28040 Madrid, Spain; m.barrachina@alumnos.upm.es; 2 Centro de Estudios e Innovación en Gestión del Conocimiento (CEIEC), Universidad Francisco de Vitoria, 28223 Pozuelo de Alarcón, Spain; a.maitin@ceiec.es; 3 Department de Matemática Aplicada a las TICs, ETSI Telecomunicación, Universidad Politécnica de Madrid (UPM), Avenida Complutense, 30, 28040 Madrid, Spain; 4 Facultad de Ciencias Experimentales, Universidad Francisco de Vitoria, 28223 Pozuelo de Alarcón, Spain; 5 Brain Damage Unit, Hospital Beata María Ana, 28007 Madrid, Spain

**Keywords:** Parkinson´s disease, motor fluctuations, sensors, motor symptoms, treatment

## Abstract

Monitoring of motor symptom fluctuations in Parkinson’s disease (PD) patients is currently performed through the subjective self-assessment of patients. Clinicians require reliable information about a fluctuation’s occurrence to enable a precise treatment rescheduling and dosing adjustment. In this review, we analyzed the utilization of sensors for identifying motor fluctuations in PD patients and the application of machine learning techniques to detect fluctuations. The review process followed the Preferred Reporting Items for Systematic Reviews and Meta-Analyses (PRISMA) guidelines. Ten studies were included between January 2010 and March 2021, and their main characteristics and results were assessed and documented. Five studies utilized daily activities to collect the data, four used concrete scenarios executing specific activities to gather the data, and only one utilized a combination of both situations. The accuracy for classification was 83.56–96.77%. In the studies evaluated, it was not possible to find a standard cleaning protocol for the signal captured, and there is significant heterogeneity in the models utilized and in the different features introduced in the models (using spatiotemporal characteristics, frequential characteristics, or both). The two most influential factors in the good performance of the classification problem are the type of features utilized and the type of model.

## 1. Introduction

Even though telemedicine was introduced many years ago, the last 20 years have been key in the development of the technologies, both in terms of software and hardware, on which telemedicine is reliant (sensors, mobile communications, cloud computing, analytics, etc.). Telemedicine is an expanding field of medicine used in healthcare-related activities, such as consultation, cooperative work between medical professionals, monitoring patients’ physiological and biometric parameters, caring for patients’ daily living conditions, and surgery (using robotics, artificial vision, and virtual reality) [1].

Parkinson’s disease (PD) is currently the second most common neurodegenerative disease, affecting 1% of the population older than 60 years [2]. According to a specialized report created by the National Institute of Health (NIH) in the United States, PD prevalence in 2018 was approximately 6 million people worldwide; by 2040, this number will have doubled.

Parkinson’s disease is characterized by motor symptoms such as bradykinesia, rigidity, and resting tremors, but also by non-motor symptoms, such as depression, apathy, or cognitive decline [3].

The administration of levodopa (L-Dopa) is the first therapeutic option for motor symptom control [4]. Depending on the degree of symptoms that a patient experiences, two states can be characterized: on state and off state. In the on state, a patient experiences an improvement in symptoms after the intake of medication. In the off state, a patient experiences a return of symptoms related to PD. As the disease progresses, patients could experience motor fluctuations, also known as “wearing-off”, which are based on the alternance between the two aforementioned states. This is one of the most challenging complications when facing treatment adjustments for PD symptomatic management. This phenomenon is reported in up to 50% of the diagnosed patients within three to five years from the start of the disease, and affects up to 80% of the diagnosed patients within 10 years from the onset of the disease [5]. In the most evolved PD cases, this occurrence is believed to be caused by basal ganglia neurodegeneration. The management of these motor fluctuations is based on various adjustments to the treatments (frequency, dosage of medication, changing parameters for brain stimulation, etc.).

The standard for PD diagnosis is based on a clinical examination of the patient by a neurologist. The patient is asked to perform specific tasks, and the severity of PD symptoms is usually graded using the Unified Parkinson´s Disease Rating Scale (UPDRS) or the Movement Disorder Society-sponsored revision of the UPDRS (MDS-UPDRS) [6]. Disease progression is measured using the Hoehn and Yahr scale (HY) [7].

Based on the evaluation and changes of these scaled scores, a physician changes and adjusts antiparkinsonian treatment to fit a patient’s personal needs. The evaluation is subjective and depends on the moment a patient was examined, with respect to the last medication intake. Due to the described fluctuating nature of the symptoms, doctors rely on patients’ perception of the intensity and severity of the symptoms during the day. This increases the complexity of the assessment since the data available for making therapeutic decisions would depend on too many subjective variables.

There are currently different wearable devices focused on identifying the characteristics of PD, which perform an assessment of motor symptoms or provide aid in disease management [8,9,10,11,12,13,14]. Furthermore, there are different novel platforms focused on the different aspects of ambulatory attention in the assessment and management of PD: detection, response, and action capabilities.

In general, technological improvements, such as greater computational power, new and different computing architectures, and greater storage capacity, have sowed the seeds for the application of AI techniques to different areas in medicine, such as diagnosis, consultation, monitoring, treatment, and prevention [15,16]. Sensors in particular, in terms of the latest trends, are becoming smaller, cheaper, and capable of wireless control, which extends their use to various applications. Therefore, the utilization of both biosignals and machine learning (ML) techniques will continue expanding over the following years, thus broadening the concepts of telemedicine and e-health and focusing on providing a personalized diagnosis or treatment to patients. 

The growing implantation of sensors for fitness tracking and smartwatches is a great source of biometric and activity data that, in the future, will require the use of applications for data processing. Most of commercially available devices rely on a suite of sensors, including combinations of cameras, inertial measurement units, gyroscopes, depth sensing, force/pressure sensors, and more to enable the user to interact with the device. According to IDTechEx, in the normal process of developing of wearable sensors, pre-designed sensors were incorporated to wearable products decades ago, and then smartphone industries provided these devices with sensors that could be made wearable; however, in recent years, many sensor types have been developed specifically with wearable products in mind [17]. This constant growth of the sensors industry may largely benefit health-related wearables to create noninvasive, portable, and cost-effective solutions. 

The purpose of this systematic review is to investigate the use of sensors in identifying the on and off states in PD patients, and to evaluate the application of ML techniques to differentiate between the on and off states. An accurate identification of a patient´s medical state may lead to successful adjustments in therapy, and may consequently have a positive impact on the quality of life of PD patients. 

## 2. Methods

### 2.1. PRISMA Statement

This review is based in the Preferred Reporting Items for Systematic Reviews and Meta-Analyses (PRISMA, http://www.prisma-statement.org) guidelines (accessed on: 10 November 2020). 

Find more information in the statement article [18] and the elaboration article [19]. 

**Figure 1:**
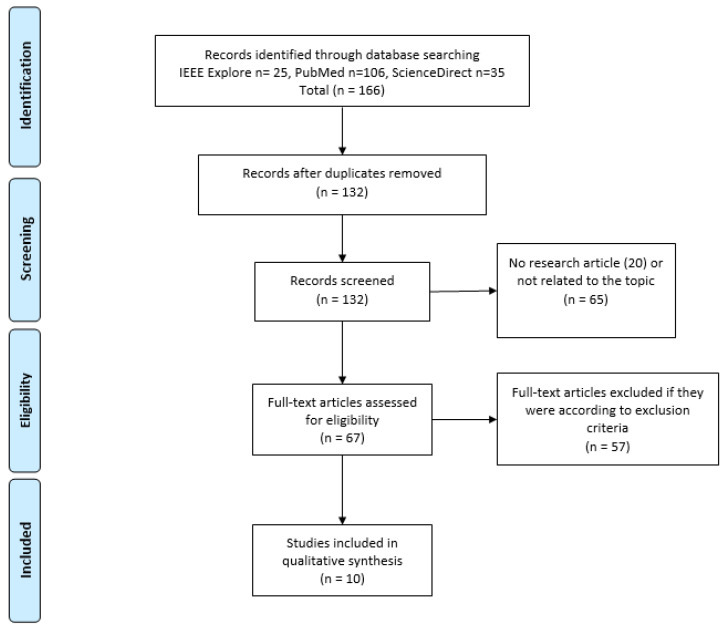
PRISMA diagram of the bibliographic review conducted.

Figure 1 shows the PRISMA flow diagram, which summarizes the search, screening, and eligibility processes carried out in this review. The precise information of each of the steps is detailed in the sections below.

### 2.2. Identification: Search Strategy and Sources

The following terms were selected: 1. Parkinson, 2. Technology, 3. Sensor, 4. Off. The proposed search term was combined using logical operators as follows: 1 AND (2 OR 3) AND 4. This combination was introduced in the following 3 databases: IEEE Explore, PubMed, and ScienceDirect. The search spanned from January 2010 to March 2021. A search was last conducted on 18 March 2021, and 166 results were obtained. 

### 2.3. Screening and Eligibility

Independently, an investigator (M.B.F) performed the initial search using the mentioned terms and evaluated the titles and abstracts of the articles found. The steps performed removed duplicates and discarded articles according to the exclusion criteria. 

The inclusion criteria were: (i) peer-review articles, (ii) human patients, (iii) patients aged older or younger than 18 years old, (iv) based on real signals or using technology, and (v) signals associated with Parkinson’s disease to identify on/off states. 

The exclusion criteria were: (i) studies that did not identify on/off states, (ii) studies using animals, (iii) articles analyzing the usage of biosignals to evaluate other motor symptoms caused by Parkinson’s disease that were different from the wearing-off phenomenon, (iv) studies not using ML techniques, (v) studies not considering signals recorded with sensors, and (vi) articles from the same group of research utilizing the same datasets 

### 2.4. Data Extraction and Analysis

After applying the inclusion and exclusion criteria, the screening process was performed. This phase was executed by two review authors (M.B.-F. and J.P.R.) in an independent manner and the activity consisted of screening the full-text articles. The final objective was to obtain a score by using the checklist following the criteria of the checklist proposed in Guidelines for Developing and Reporting Machine Learning Predictive Models in Biomedical research [20]. The mentioned checklist is composed of 12 different reporting items that should be included in a research article to assure its quality. Below is a summary of the items utilized for the quality assessment:

- Items 1, 2: focused on reviewing the title and the abstract.
- Items 3, 4: evaluate the information provided in the introduction.
- Items 5, 6: evaluate the description and completeness of the dataset and the main method/s analyzed in the research.
- Item 7: this point evaluates the data pre-processing method, if any, and, in general, any step to prepare the data for analysis.
- Item 8: this item is related to the steps that form the predictive model.
- Item 9: this is focused on evaluating the performance on the evaluated model.
- Items 10, 11 and 12: these items are related to the quality of the discussion. They evaluate the existence and quality of the clinical implications, the limitations of the study, and unexpected results.

Two independent evaluators (M.B.-F. and A.M.M.) followed the checklist mentioned before evaluating each selected article. Furthermore, to score the consensus between the evaluators, the kappa value was calculated. The main purpose of this procedure was to generate an objective assessment for each content´s article to make them comparable to one another. Therefore, the quality evaluation is also indicated in the Section 3.

### 2.5. ML Techniques

This section provides an initial conceptual framework for the main types of ML technique. 

Supervised algorithms utilize labeled data to train the algorithms; however, unsupervised algorithms discover information from the input data. As can be seen in Table 1, the main purpose of the supervised algorithms is classification or regression (or both); meanwhile, the objective of the unsupervised algorithms is identifying unknown patterns in the data.

## 3. Results

### 3.1. Eligibility According to PRISMA Flow Diagram

According to the PRISMA methodology, a flow diagram is shown in Figure 1. A total of 166 studies were initially identified in the search process. After the removal of duplicate studies (34), the titles and abstracts of 132 articles were screened and 65 irrelevant records were excluded, as they were not related to the evaluated topic (40), to reviews (5), or were not peer reviewed (20 articles from proceedings and conferences). Consequently, 65 studies were removed in this step, leaving a total of 67 of articles, which were submitted to the eligibility process. The criteria for inclusion and exclusion were applied. As a result of this phase, eight articles were excluded for not using ML techniques and three articles did not use sensors; however, 46 articles focusing on Parkinson motor symptoms (gait or bradykinesia) did not examine on/off states. The sum of all these types of article resulted in a total of 57 exclusions, leaving us with a total of 10 studies that met the defined inclusion criteria. Of the included articles, the main characteristics related to sensors utilized, study goal, classifier used, and performance obtained are shown in Table 2. 

### 3.2. Analysis of the Quality of the Articles

**Figure 2:**
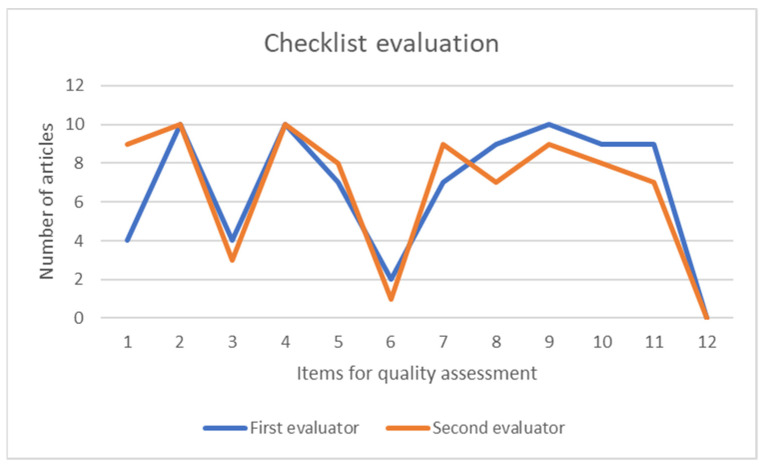
Plot of the number of selected articles that satisfy each of the items of the checklist used.

The quality of the articles was evaluated using the checklist presented before, with the main purpose of comparing the content of the publications. The first evaluator provided an average value of 8.1 ± 1.59 out of 12 for the 10 articles, whereas the second evaluator determined an average assessment of 8.1 ± 1.52 out of 12. With the main purpose of assessing the concordance between both evaluations, the kappa (k) value was calculated. This value reflects the effect of a chance on an agreement in the observation, obtaining a value of k = 0.66 and showing a moderate level of agreement among the evaluators (a value greater than 0.7 means a high level of agreement among the evaluators, a value between 0.5 and 0.7 means a moderate level of agreement, and a value lower than 0.5 means a low level of agreement) [21]. Figure 2 shows a simple plot, with information regarding the number of articles and punctuation for each checklist item. 

Evaluating the content of the articles included in this review, Table 2 shows the summary of the main characteristics for each study. These data include the title of the study, the authors, the publication year, the study country, the study design, the sample size of patients, disease stage, the sensors used, and the features utilized in the classifier, as well as the the classifier utilized and the result parameters in the identification of the on/off states. If a full paper was not found online, articles were requested directly from authors. 

**Table 2:** (**a**) Characteristics of included studies (I). Acronyms: IMU—inertial movement unit; SVM—support vector machines; kNN—k-nearest nearest neighbor; CNN—convolutional neural networks; UPDRS—unified Parkinson’s disease rating scale; ANN—artificial neural network; H and Y—Hoehn–Yahr; F—female, M—male. (**b**) Characteristics of included studies (II). (**c**) Characteristics of included studies (III).

(a)											
Title	Authors	References	Country	Publication Year	Sample Size	Sex (F/M)	Stage (UPDRS or H&Y)	Sensor	Features	Classifier	Performance Indices and Outcome
A Kinematic Sensor and Algorithm to Detect Motor Fluctuations in Parkinson Disease: Validation Study Under Real Conditions of Use	Rodriguez-Molinero, A. et al.	[22]	Spain	2018	23	7/16	21 ± 16 UPDRS	IMU	Spatiotemporal gait	Own machine learning algorithm	Accuracy (92.2%)
A Supervised Machine Learning Approach to Detect the On/Off State in Parkinson’s Disease Using Wearable Based Gait Signals	Aich, S. et al.	[23]	South Korea	2020	20	8/12	15.8 ± 10.13 UPDRS	Accelerometer	Statistical features + spatiotemporal gait features	Random forest, kNN, SVM and naïve Bayes	Accuracy (96.72%), recall (97.35%), precision (96.92%)
A Treatment-Response Index from Wearable Sensors for Quantifying Parkinson’s Disease Motor States	Thomas, I. et al.	[24]	Sweden	2017	19	5/14	Advanced stage	Accelerometer and gyroscope	Spatiotemporal features	SVM, decision tree, random forest, linear regression	Classification accuracy (89%, 74%, 84%, 81%)
(**b**)											
Analysis of Correlation between an Accelerometer-Based Algorithm for Detecting Parkinsonian Gait and UPDRS Subscales	Rodriguez-Molinero, A. et al.	[25]	Spain, Italy, Israel, Ireland,	2017	75	27/48	15 ± 13 UPDRS	IMU	Spatiotemporal gait features	SVM	Correlation (rho −0.73; *p* < 0.001)
Assessing Motor Fluctuations in Parkinson’s Disease Patients Based on a Single Inertial Sensor	Pérez-López, C.	[26]	Spain	2016	15	5/10	2.66 H&Y	IMU	Spatiotemporal, frequential gait features	hierarchical algorithm	Specificity (92%), sensitivity (92%)
Assessment of response to medication in individuals with Parkinson’s disease	Hssayeni, M.D. et al.	[27]	United States	2019	19	5/14	14 ± 8 UPDRS	Gyroscope and accelerometer	Spatiotemporal, frequential gait features	SVM	Accuracy (90.5%), sensitivity (94.2%), specificity (85.4%)
High-Resolution Motor State Detection in Parkinson’s Disease Using Convolutional Neural Networks	Pfister, F.M.J. et al.	[28]	Germany	2020	30	10/20	21.6 ± 15.3 UPDRS	IMU	Spatiotemporal gait	CNN	Sensitivity (64%), specificity (89%)
(**c**)											
Multilevel Features for Sensor-Based Assessment of Motor Fluctuation in Parkinson’s Disease Subjects	Ghoraani, B. et al.	[29]	United States	2019	19	5/14	UPDRS: 14 ± 8	Gyroscope	Time-domain features, frequency-domain features	SVM	Accuracy (83.56%), sensitivity (78.51%), specificity (92.02%)
Unsupervised home monitoring of Parkinson’s disease motor symptoms using body-worn accelerometers	Fisher, J.M. et al.	[30]	United Kingdom	2016	34	Not specified	H&R I-IV	Accelerometer	Temporal features	ANN	Sensitivity (51%), specificity (87%)
Validation of a portable device for mapping motor and gait disturbances in Parkinson’s disease	Rodriguez-Molinero, A. et al.	[31]	Spain	2015	35	8/27	H&Y III	Accelerometer	Frequential and spatiotemporal parameters	SVM	Sensitivity (96%), specificity (94%)

### 3.3. Analysis of the Selected Articles

Evaluating the number of articles dedicated to the topic analyzed in the period 2010–2021, it is possible to see that the articles are concentrated in the period 2015–2021, with a stabilization in the number of articles, as they are distributed between one or two (maximum) articles per year. 

Evaluating the number of patients in the selected studies, it is important to highlight that most of the studies (nine out of 10) included fewer than 50 patients, where the average value was 28.9 ± 17.6. The articles do not state in the analysis or discussion whether the number of patients selected was appropriate for the classification problem in the scope of the study. Regarding the age of patients, PD patients included in the analysis were between 44 and 78 years, with a mean value oscillating around 60 years, which is coherent with the expected age of incidence of the disease. The total number of patients evaluated in the selected studies is 289, with 27.7% being female patients, 60.6% being male patients, and 11.7% not indicating their sex. The duration of the disease in the patients evaluated is specified in nine out of 10 studies in the scope of this review, with a mean duration oscillating at around 10 years. When the degree of the progression of the disease was considered, six out of 10 of the evaluated articles indicated the status of the patients according to the UPDRS scale, with three of the studies showing the status of patients according to the Hoehn–Yahr (HY) scale. Only one of the articles evaluated did not mention the specific stage of the disease, and only mentioned an advanced stage of the illness. 

With respect to pre-processing, there is no common procedure for cleaning captured signals, so this varied between articles (some of the articles did not include those details and others used a specific filter to clean the captured signals). This point makes the quality assessment of the datasets difficult to evaluate. In particular, four of the articles performed signal pre-processing by minimizing signal noise using different types of a Butterworth filter, and the remaining articles did not specify the cleaning process, assuming no alterations in the signal’s capture. 

Related to the input characteristics, the features analyzed from the captured signals are very different between the articles. In particular, five articles utilized spatiotemporal characteristics, three articles combined spatiotemporal and frequency characteristics, one article combined statistical and spatiotemporal features, and one article utilized only frequential characteristics. 

One important factor in this review is to analyze the technology utilized in the selected studies. According to the evaluated studies, the main technology used in 40% of the articles analyzed is an inertial sensor (IMU is a device with an accelerometer, a gyroscope, and sometimes a magnetometer). The combination of an accelerometer and a gyroscope is used in 20% of the cases. Only an accelerometer is utilized in 30% of the articles, and only a gyroscope is utilized in 10% of the studies. These results show those devices are a trustworthy, low-cost solutions for assessing movement symptoms in PD patients. 

It is relevant that the on/off state is a binary variable, but the reality is that patients experience a smooth change between one state and the other one. The reviewed studies utilized different controlled protocols to record the data; once in the on state (when taking the medication) and then in the off state (an artificially induced prolonged withdrawal time of the patient’ normal medication).

#### 3.3.1. Types of Models Considered

**Figure 3:**
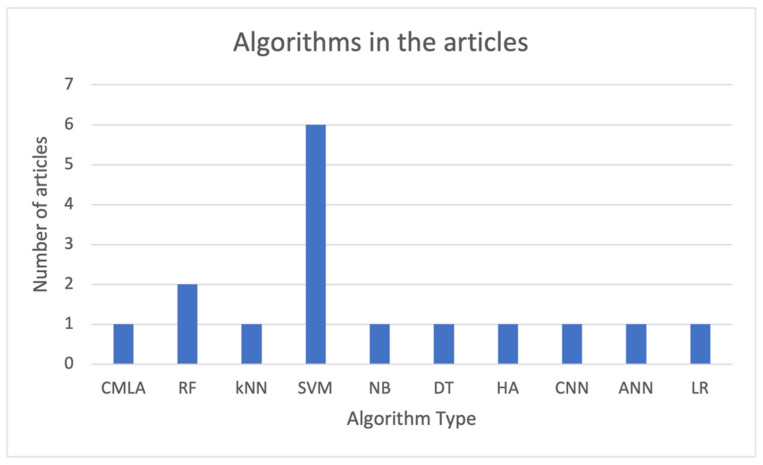
Diagram bar with the type of algorithms utilized. Acronyms: SVM—support vector machines; KNN—k-nearest neighbors; DT—decision tree; CNN—convolutional neural network; RF—random forest; LR—linear regression; NB—näive Bayes; HA—hierarchical algorithm; ANN—artificial neural network; CMLA—customized machine learning algorithm.

In this review, it is important to evaluate the variety of models used. Figure 3 shows a bar chart with the different models from the analyzed articles. 

Related to the machine learning models shown in Table 2, the 10 selected articles utilized a total of 10 different ML techniques to approach the classification problem, highlighting that there are articles using more than one technique (as shown in Figure 2). More precisely, there are two articles in which four different ML methods are tested and compared; however, there are eight articles in which a unique method is utilized. 

Support vector machine (SVM) algorithms play an important role in the studies, as they are utilized in six out of 10 articles evaluated. Random forest (RF) is utilized in two different articles, and the rest of the algorithms are used on one article each. Considering the type of processing linked to the models, decision tree (DT) and RF algorithms can be considered symbolic models, while the remaining ones are categorized into the subsymbolic group. According to Figure 2, most of the models utilized in the evaluated articles belong to the subsymbolic category. 

Finally, to conclude this initial analysis, it is important to mention that there is huge heterogeneity between the models utilized in the different articles evaluated, and they each have special considerations when comparing results obtained in the classification problem. The results obtained in each article will be discussed in the subsequent sections of this review. 

#### 3.3.2. Type of Data Collected

As can be seen Table 3, the selected articles considered two types of activity to collect the data. Consequently, the articles were divided into two different categories based on how the type of activity utilized acquired the data. On the one hand, the daily living activities group corresponds to articles [21,25,26,27,28], in which the data evaluated have been collected during normal daily life activities. On the other hand, the specific activities group corresponds to articles [22,23,25,30], which recorded the data by performing specific activities during concrete sessions. Moreover, there is one specific article using both types of activity [29].

For each of these articles, the model considered, the classification results obtained, the characteristics introduced, and the type of signal processing are shown in Table 3. 

##### Daily Living Activities

The daily living activities group contains five articles which considered different algorithms and sensors and utilized different features from them. The articles exhibited different sensors (inertial sensor, gyroscope, and combination of gyroscope and accelerometer). On the one hand, the classifier utilized in 40% of the cases is SVM, obtaining an average accuracy of 86.78%, an average sensitivity of 86.355%, and an average specificity of 88.71%. Those articles use a filter to clean the input signal coming from the sensors, specifically a bandpass filter (with normalized pass frequency in the band 0.5–1.5 Hz). Those studies utilized both time domain features and frequency domain features. On the other hand, the other 60% cases use a different classifier such as a customized algorithm [22], a CNN algorithm [28], or a hierarchical algorithm [26], but all of them use an accelerometer as the sensor to capture the data. There is only information for the CNN algorithm in relation to the signal cleaning, i.e., using a two direction Butterworth filter. The accuracy for those articles is only provided for [22], reaching a value of 92.2%. The average specificity for the other remaining two articles is 90% [26,28]. 

Finally, in this group, the evaluation of the quality criteria performed by the two evaluators provide the following values: first, an average value of 8.4 ± 1.14 out of 12 for the first evaluator and, second, a value of 8.6 ± 1.51 out of 12 for the second evaluator. The kappa value obtained for the articles included in this group was 0.74, and was consequently higher than the value obtained when analyzing all the evaluated articles. Therefore, the level of agreement between the evaluators is higher when considering the category of daily activities. 

##### Specific Activities

This category evaluated four articles [22,23,24,30]. Half of those studies used more than one model to solve the classifying problem, and the remaining ones only used one specific classifier. The common characteristic between them is that they all used the SVM classifier. All of them used an accelerometer as the sensor to capture the data, and there was only one that used a combination of both an accelerometer and a gyroscope [24]. The articles utilizing more than one classifier [22,23] used a filter to decrease the level of noise in the signal by using a low pass Butterworth filter [23] or an approximate entropy method for motion removal [24]. There is no common metric between the studies of this group, and the accuracy, sensibility, and specificity between the utilized metrics provide results. There is only one article [31] that utilizes frequency parameters as features instead of spatiotemporal features in this category [22,23,24]. 

To conclude this group, the evaluation of the quality criteria, according to the checklist introduced before, received an assessment of 8.25 ± 2.06 out of 12 by the first evaluator and 8 ± 1.41 by the second evaluator. The kappa value for this group was 0.44, showing a slightly lower value than the one obtained in the previous group and in the global set of articles, indicating a lower level of agreement between the evaluators. 

##### Combination of Both (Daily Activity + Specific Activities) 

Finally, there is a third category that is represented only by one article [30], and which uses ANN as a main classifier, an accelerometer as the sensor to capture the data, and temporal features to obtain a sensitivity of 51% and a specificity of 87%. This specific case combines daily life activities with specific activities performed in a controlled environment. 

## 4. Discussion

PD is a disease in which motor complications have an important impact on a patients´ quality of life and represent a major issue in treatment adjustment. The purpose of this systematic review was to investigate the usage of sensors in identifying the on and off phenomena in PD patients and to evaluate the application of ML techniques to differentiate between the on and off states. The evaluation found 10 different studies in the literature review that analyzed the usage of sensors to detect the on and off medication status. It can be seen from the included articles that this field is still emerging, with the oldest article being published in 2015. 

Implementing applications to help clinicians in the management of Parkinson’s patients has increased in recent years, especially the utilization of wearable devices which are key for early diagnosis and the monitoring of motor symptoms. The purpose of these devices is to improve a patient´s quality life by having a constant medical control, with the objective of providing accurate and personalized treatment. According to an article from the Movement Disorder Society (MDS) Task Force on Technology [24], there are different challenges to be solved when considering improving a PD patient´s life, including solving the need for monitoring non-motor symptoms, being able to limit the sensors used to monitor a patient´s motor symptoms, the actual discrepancy between clinical needs and research, a lack of compatibility among wearable systems, and the limitation of available analytical methods or practical limitations in user engagement [32]. 

Several platforms have been developed in the past to control Parkinson’s disease (PD) symptoms. An example is the REMPARK project [33], which is based on a wearable monitoring system that can monitor patient motor status and evaluate the on/off status with high precision. Another relevant example is known as the “HELP” system, which is is focused on optimizing treatments and improving the life quality of patients with PD by estimating PD symptoms and adjusting the dose of medication to reduce them [34]. Another great example of the platforms created in relation to PD is an innovative project funded by the European Union research and innovation program, Horizon 2020, which is related to identifying Parkinson’s Disease, called i-PROGNOSIS [35]. The main purpose of the mentioned project is to develop early and unobtrusive Parkinson´s disease detection tests, primarily based on the interaction of users with daily technological devices (largely by monitoring the patient’s movements and mood and with direct communication with their doctors). 

Related to the global distribution of the articles, most of the studies were conducted in Spain and the United States (six out of the ten studies were analyzed, with four performed in Spain and two performed in the United States). The remaining studies were conducted in South Korea, Sweden, Ireland, Israel, Italy, Germany, and the United Kingdom, showing a global interest in engaging with the objectives of this review. 

The quality of the selected articles was assessed in this review using the checklist of the guidelines for developing and reporting machine learning (ML) predictive models in biomedical research. The average values obtained (8.1 ± 1.59 out of 12 and 8.1 ± 1.52 out of 12) indicate the high-quality level of the articles in the review. Another important fact to mention is a kappa value of 0.66 among the two independent reviewers, showing a moderate level of agreement between the evaluators when assessing the quality of the articles in this study. It is important to mention that both evaluators agree that the less fulfilled items were 6 and 12, clearly related to the definition of the prediction problem and the unexpected results, respectively. Unexpected results could appear after more research and new articles related to the topic are published. 

It is important that good results were obtained in the evaluated articles, even when signals were captured in two totally different conditions. In both situations, the most used technology is an accelerometer, an accessible and minimally invasive tool currently available. 

The utilization of biosignals along with ML techniques has grown in recent years, and has been applied to several different diseases. Related to PD, various studies show the importance of those techniques for identifying PD using electroencephalography (EEG) [36] or for diagnosing and assessing PD using inertial sensors or video signals [37]. There are other diseases being assessed that utilize the same approach; for example, identifying atrial fibrillation using an electrocardiogram (ECG) and applying ML techniques to identify potential alterations [38], or diagnosing Alzheimer’s disease using the ML algorithms by processing sensor movement data from patients [39,40]. 

To conclude, it is important to discuss how the features utilized and the cleaning process of the signal can directly influence the efficiency of the models used. As is shown in Table 2 when comparing articles [22,23,24,26,28,30], six out of 10 articles use SVM, resulting in an accuracy of 93% [23], 89% [24], 90.5% [27], and 83.56% [29], or they show the results in terms of correlation, with a rho value of −0.73 [25] or sensitivity [31]. From that information, it is possible to see that the highest accuracy is obtained when using input information for the classifier, as well as a combination of the statistical features and the spatiotemporal information of the signals captured [23]. Considering the good results obtained in the mentioned article, another important point is that the utilization of the low pass Butterworth is a useful method to clean the input signals coming from the sensors when utilizing ML techniques for classification. Therefore, it can be inferred that the utilization of a Butterworth filter for cleaning the input signal coming from the sensors and using features from the statistical information of the captured data and the spatiotemporal information are decisive for obtaining a better performance in the classification problem. 

For the most novel techniques utilized in the articles evaluated, CNN and ANN in this case, there is no conclusion regarding the best filter to use in [24]; the filter utilized is not specified and the sensitivity obtained is only 51%. That said, in [28], the sensitivity obtained rises to 64% with a two direction Butterworth filter.

Therefore, in order to develop an accurate model that can classify the on/off states associated with PD, it is essential to take into account both the input parameters to the classifier and the signal cleaning methods. 

In general, current publications and records exploring the usage of sensors to identify the wearing-off phenomenon suggest that the results are optimistic, and that the utilization of wearable devices could provide an improvement in the management of the PD symptoms. The evidence suggests that using technology is acceptable for clinical applications, according to the accuracy obtained, even though there can be different barriers for patients to engage with this approach. The evaluated studies showed good results in the classification problem, in some cases reaching up to 90% accuracy. After a deeper analysis of the models and features utilized in the studies, it can be concluded that the types of feature utilized in the model, as well as the cleaning process, are key in attaining good performance in the classification problem. Moreover, there are multiple factors that influence the utilization of this technology by patients, such as the health status of the user, its usability, convenience, accessibility, perceived utility, or motivation [41]. 

In the technological environment, there are numerous challenges to overcome that have not been assessed in the included articles, such as those related to privacy/confidentiality issues, adequate infrastructure, user identification, etc. 

## 5. Conclusions

The role of machine learning techniques is growing in relation to solving complex problems. Those techniques are being applied to the management of different diseases such as heart disease, diabetes, breast cancer, or Parkinson’s. This review focused on evaluating the utilization of wearable devices to identify the on/off states affecting PD patients using ML techniques. Three major results stand out from this review. First, the usage of a low-cost technology has shown itself to be enough to predict the on/off state of a PD patient, and this fact is relevant when considering how to improve a patient´s quality of life. Second, although there are still a limited number of articles in the literature applying ML techniques to the identification of the on/off states, research into this topic has stabilized; in recent years, the number of articles on the subject is homogeneous, showing that the field is developing but the speed of research has slowed down. 

For future research, it is important to extend the usage of ML techniques for non-motor symptom fluctuations’ detection, as the monitoring of symptoms such as pain and dysautonomic and neuropsychiatric symptoms is a next step in the control of the disease. This approach will help to complete the assessment and provide a more accurate treatment adjustment. Moreover, the results presented were obtained in clinical trials; however, it is necessary to perform more testing to evaluate results in real patient populations with different levels of disease severity and in different environments, to overcome challenges, and to extend the results obtained and potentially establish the usage of technology to assess and treat PD in a generalized way. In future research, it is crucial to include the identification of different factors that could potentially affect the engagement of technological solutions for managing Parkinson’s from different perspectives, including health professionals, care givers, and patients. This research will help to identify the challenges that those users could face and try to propose solutions from different points of view. 

The future of the field will be related to increasing research and investment, with the main purpose of extending clinical trials of the applications to real life by using existing devices used every day, such as smart phones or smart watches, facilitating access to the benefits that those devices could bring to a PD patient’s life.

## Figures and Tables

**Table 1:** Summary of main ML technique types.

ML Type	Purpose	Typical Algorithms	Description
Supervised algorithm	Classification	Naïve Bayes, logistic regression, support vector machines	The main purpose of these algorithms is to classify data into the different predefined classes
	Regression	Linear and non-linear regression	The main purpose of these algorithms is to find the relation between different variables
	Both	Decision trees, random forest, k-nearest neighbors, neural networks	These have classification properties but also the ability to find the relation between different variables
Unsupervised algorithm	Clustering	K-means, neural networks, hidden Markov model	The main purpose of these types of algorithm is to discover groups in the input data

**Table 3:** Summary of the results, type of features introduced to the model, year of publication, and main results obtained. Acronyms: BW—band width; SVM—support vector machines; kNN—k-nearest neighbors; NB—Naive Bayes; DT—decision tree; RF—random forest; LR—linear regression; UDPRS—unified Parkinson’s disease rating scale; CNN—convolutional neural networks; ANN—artificial neural network; FIR—finite impulse response.

Refs	Year	Features	Cleaning Method	Results	Classifier	Perf. Indicator
[22]	2018	Spatiotemporal characteristics	Not specified	92.20%	Own machine learning algorithm	Accuracy
[23]	2020	Statistical features + spatiotemporal features	Low pass BW filter	RF: 96.72%, 97.35%, 96.92%; SVM: 93%, 02%, 93%; KNN: 86%, 84%, 85%; NB: 88%, 86%, 85%	Random forest, kNN, SVM, and Naive Bayes	Accuracy, recall, precision
[24]	2017	Spatiotemporal features	ApEn method for motion removing	SVM:0.89, DT: 0.84, RF: 0.81, LR: 0.74	SVM, decision tree, RF, linear regression	Classification accuracy
[25]	2017	Spatiotemporal features	Not specified	Correlation between the algorithm outputs gait status (rho −0.73; *p* < 0.001)	SVM	Correlation with UPDRS-III
[26]	2016	Spatiotemporal features + frequency features	Not specified	92%, 92%	Hierarchical algorithm	Specificity and sensitivity
[27]	2019	Spatiotemporal + frequential features	Bandpass FIR filter	90.5%, 94.2%, 85.4%	SVM	Accuracy, sensitivity, specificity
[28]	2020	Spatiotemporal features	Two direction BW filter	64%, 89%	CNN	sensitivity, specificity
[29]	2019	Time-domain features and frequency-domain features	Bandpass filter	83.56%, 78.51%, 92.02%	SVM	Accuracy, sensitivity and specificity
[30]	2016	Temporal features	Not specified	51%, 87%	ANN	Sensitivity, specificity
[31]	2015	Frequency parameters (spectral power)	Not specified	96%, 94%	SVM	Sensitivity, specificity

## Data Availability

No new data were created or analyzed in this study. Data sharing is not applicable to this article.
